# Subsoiling during summer fallow in rainfed winter-wheat fields enhances soil organic carbon sequestration on the Loess Plateau in China

**DOI:** 10.1371/journal.pone.0245484

**Published:** 2021-01-27

**Authors:** Huiyu Zhang, Zhiqiang Gao, Jianfu Xue, Wen Lin, Min Sun

**Affiliations:** College of Agriculture, Shanxi Agricultural University, Taigu country, Shanxi, China; Emory University, UNITED STATES

## Abstract

Scientific management of the soil organic carbon (SOC) pool, e.g., through a reasonable tillage system, is a potential way to mitigate global climate change. There is scarce information about the effect of tillage during the summer fallow period on the SOC pool in rainfed winter-wheat fields. The present study was designed to evaluate the effects of tillage practices, i.e., plow tillage (PTF), subsoiling (STF) and no tillage (NTF), during the summer fallow period on SOC sequestration in winter-wheat fields in the rainfed area of the eastern Loess Plateau of China. The SOC, mineral-associated organic carbon (MOC), permanganate-oxidizable organic carbon (POxC) and particulate organic carbon (POC) concentrations were determined after four years of tillage implementation during the summer fallow period. Our results showed that in comparison to the adoption of NTF, the adoption of STF significantly increased POxC, POC and MOC concentrations by 56.6–111.2%, 45.7–118.7% and 26.2–29.4%, respectively, at the 10–20 and 30–40 cm soil depths before sowing (*P* < 0.05). The POxC and MOC concentrations under STF at depths of 0–10, 10–20, 20–30 and 30–50 cm were significantly greater than those under PTF and NTF after harvesting (*P* < 0.05). In addition, the SOC concentration and SOC stock under STF were significantly greater than those under NTF at the 0–10, 10–20, 20–30 and 30–40 cm soil depths before sowing and after harvesting (*P* < 0.05). Furthermore, in comparison to PTF and NTF, STF resulted in significantly higher SOC stocks by 12.0–25.3% and 7.1–19.2% than PTF and NTF, respectively, in the 0–10, 0–20, 0–30, 0–40 and 0–50 cm soil profiles at harvesting (*P* < 0.05). In summary, the adoption of STF could be beneficial to the management of the SOC pool in the 0–50 cm soil profile in the rainfed area of winter-wheat on the Loess Plateau of China.

## Introduction

Soil organic matter (SOM) plays a critical role in soil fertility maintenance and soil moisture retention, which indirectly contributes to the crop productivity and food security. Soil organic carbon (SOC), as the most important component of SOM, not only affects soil quality and crop yield, but also has a crucial impact on mitigating climate change [1]. Drylands account for 47.2% of the global land area, and 15.5% of the SOC in the 0–1 m soil profile [2], which possesses a large carbon sequestration potential [3]. Under a scenario of high emissions of greenhouse gases, the area of global drylands has been estimated to expand. By the end of the 21st century, the area of global drylands will reach approximately 50% of the area of global land [4]. Dryland agriculture is facing more severe challenges. Increasing SOC sequestration through effective management practices is of great significance for mitigating climate change and the sustainable development of dryland agriculture.

Agricultural management practices such as tillage, fertilization, and residue incorporation could strongly influence the soil microenvironment and exert an impact on SOC turnover and sequestration. Generally, plow tillage accelerates the SOC decomposition due to the damaged soil structure, which directly decreases the SOC stock [5,6]. Therefore, conservation tillage is receiving increased attention. It has been reported that conservation tillage, which involves no tillage or reduced tillage reduces soil disturbance, effectively prevents soil being exposed to air, decreases mineralization of SOC and could affect SOC stock by crop-residue mulching [7].

Tillage practices affect SOC dynamics and reduce the global SOC stock by 0.06% per year [8]. Plow tillage has been considered an important factor in causing a decline of SOC stock. A twelve-year study in northeastern China showed that the SOC stock increased under no tillage but decreased under plow tillage compared to that under original soils [9]. Similarly, another experiment showed that the SOC stock in the 0–60 cm soil profile increased under no tillage with straw mulching and subsoiling with straw mulching compared with that under crop residue removal [10]. However, the SOC stock in the topsoil of southern Finland was lower after thirty years of reduced tillage than that under the conventional tillage, which may be related to the already high level of SOC concentration [11]. Stewart, et al. [12] collected soil samples derived from forty-eight farmlands that adopted thirty years of continuous no tillage, and they showed that there was no evidence of SOC saturation with statistical modeling. Differences in climatic conditions, soil conditions, and management systems may cause differences in the distribution of and variation in SOC stocks.

No tillage can improve the SOC concentration in the surface layer [13]; however, it is still controversial whether the SOC concentration in the deeper soil layers will be affected by long-term tillage [14,15]. The results of a twenty-two-year tillage experiment showed that the SOC concentration under no tillage at a 0–20 cm depth increased by 30% compared with that under plow tillage [16]. A six-year long-term experiment with reduced tillage showed a 19% increase in the SOC concentration at the 0–10 cm soil depth, while the SOC concentrations at the 10–20 cm depth remain constant under reduced and plow tillage [17]. Plaza-Bonilla et al. [18] reported that the SOC concentration under no tillage was reduced by approximately 7–16% at a soil depth of 5–40 cm compared with that under plow tillage.

As the change in SOC concentration often occurs slowly, it is difficult to show the change in SOC under short-term tillage practices [19]. Therefore, researchers are focusing more on labile SOC fractions due to their higher sensitivity to tillage. Long-term studies indicate that the concentrations of particulate organic carbon (POC) and permanganate-oxidizable organic carbon (POxC) at the 0–20 cm soil depth decrease under plow tillage [19,20]. In contrast, other studies show high POC or no change in POC under plow tillage compared with no tillage or reduced tillage [14]. No tillage increased the POxC concentration at the 0–10 cm and the POC concentration at the soil surface at 2.5 cm; however, the effect on the subsoil was inconsistent and mainly varied in terms of soil texture, residue inputs and previous management [21]. Currently, information on the impact of tillage during the summer fallow season in drylands on POxC and POC concentrations is scarce.

As a relatively recalcitrant SOC fraction, mineral-associated organic carbon (MOC) could reflect the effect of SOC sequestration because of a high amount of clay and silt particles and high stability [22]. In comparison with plow tillage, no tillage for six years resulted in a 5.3 Mg ha^-1^ increase in the SOC stock at the 0–10 cm soil depth, and 38% of the SOC accumulation occurred in the MOC fraction [23]. Ferreira et al. [24] shows that the MOC concentration under no tillage in the 0–5 cm soil layer was lower than that under plow tillage, whereas Plaza et al. [25] reports that the MOC concentration was16% higher in no tillage plots than in plow tillage plots. However, Blanco Moure et al. [26] showed that no change in MOC concentrations under tillage practices occurred under rainfed Mediterranean conditions. There is scarce information about the effect of tillage during the summer fallow season on the MOC concentration in drylands.

Wheat *(Triticum aestivum L*.*)* is a dominant staple crop in China. Dryland wheat plays a crucial role in agricultural production on the Loess Plateau, the yield of which is limited by water shortages and soil infertility, and a large yield fluctuation occurs frequently [27]. It is important to effectively conserve soil water, to enhance soil carbon sequestration and to obtain stable and high yields for the sustainable production of dryland wheat. The implementation of tillage practices (plow tillage and subsoiling tillage) over the summer fallow season can conserve soil moisture for winter-wheat production in drylands [28]. However, the effects of the SOC pool have not been well studied. Therefore, exploring SOC sequestration and studying SOC fractions under different tillage practices in winter-wheat fields would provide useful information to manage the SOC pool in dryland areas. In this study we evaluated the effect of different tillage practices during summer fallow on SOC sequestration and changes in the distribution of SOC, POxC, POC and MOC in the 0–50 cm soil profile.

## Materials and methods

### Study site

Field-tillage experiments were conducted at the experimental site of Shanxi Agricultural University in Wenxi, Shanxi Province (111°28’E, 35°35’N). This area is located on the eastern Loess Plateau in China and is characterized by a warm temperate monsoon climate that receives 490 mm in mean annual precipitation and experiences a 12.6°C average annual temperature, 1838.9 mm average annual potential evapotranspiration, and 2461 hours of average annual sunshine hours. In the primary cropping system in the drylands of the Loess Plateau region, winter-wheat is planted as a single crop in late September and is harvested in early June of the next year. The bare field is left over the summer fallow period from early June to late August.

### Field management and experimental design

The tillage experiment began in 2011 and the data were collected in 2015. Treatments involved three tillage practices, i.e., no tillage (NTF), deep plow tillage (PTF), and subsoiling (STF), during the summer fallow, and the treatments were arranged in a completely randomized block design with three replicates for each treatment. The area of each plot was 150 m^2^ (50 m × 3 m). Rotary tillage was adopted before the experiment was established in 2011. During each year in 2011–2015, winter-wheat was sown in October and harvested in June of the following year. The 20–30 cm stubble from wheat crop was left in the field after harvesting wheat previously in early June. Tillage practices were conducted a few days after heavy rain from early July to mid-July, where the plowing depth was 25–30 cm for PTF, whereas for STF, the plots were subsoiled at a 30–35 cm soil depth. Commercial organic fertilizer, which contains more than 45% of organic matter, was applied at a rate of approximately 600 kg ha^-1^ for each plot before the tillage operation. Rotary tillage (15–20 cm depth) was performed at the end of August to control weeds in all three tillage treatments. The basic soil properties of the 20 cm soil layer in the experimental field were determined before starting experiment in 2011 and the results showed that the soil contained 8.72 g kg^-1^ of organic matter, 19.87 mg kg^-1^ of available phosphorus, 40.16 mg kg^-1^ of available nitrogen and 0.78 g kg^-1^ of total nitrogen.

Before sowing, approximately 600 kg ha^-1^ of compound fertilizer (N:P_2_O_5_:K_2_O = 20:20:5) was applied to all plots. Rotary tillage at a depth of 15–20 cm was carried out again in all three tillage treatments to sow winter-wheat. For all plots, the seeds were drilled along the sides of ridges, and the ridges were covered with plastic film. Ridging, sowing and mulching were performed at the same time. The ridges were a circular arc shape with a 40 cm width and a 10 cm height and were mulched with transparent plastic film. The thickness of the plastic film was 0.01 mm, and it was 400 mm wide. A seeder was used to dig a furrow that was 10 cm wide and 5 cm deep. The seeds were sown in furrows with a 20 cm spacing. The plastic film was removed after 10–15 days of anthesis. Pesticides were used to control disease and insect infestation according to the local standard for winter-wheat cultivation. The cultivar used in this study was the semi-winter-wheat variety ‘Jinmai 47’. Planting with a 97.5 kg ha^-1^ of seeding rate occurred on 26 September 2015, and harvesting occurred on 10 June 2016.

### Sample collection and analysis

Five soil samples were taken from each plot before sowing (26 September 2015) and after harvesting the winter-wheat (10 June 2016). The intersection of two diagonal lines in the rectangle plot was used as the center point for soil sampling, and the other four points of soil sampling occurred along the two diagonal lines, at the same distance (50.09 m). Soil samples were sampled using a core sampler with a 5 cm diameter. The five soil samples were collected from a 0 to 50 cm depth in 10 cm increments for all plots. The five cores were thoroughly mixed to make a composite soil sample. Soil samples were stored in Ziploc bags and brought back to the laboratory for analysis. The concentrations of SOC (*C*_*SOC*_) and its fractions were determined, including soil permanganate-oxidizable organic carbon (*POxC*) concentration (*C*_*POxC*_), soil particulate organic carbon (*POC*) concentration (*C*_*POC*_), and soil mineral-associated organic carbon (*MOC*) concentration (*C*_*MOC*_). Undisturbed soil samples were also simultaneously collected to analyze soil physical characteristics, including bulk density, moisture content, porosity and aggregate properties. In addition, available N, available P, available K, and β-glucosidase and urease activities in the soil samples were determined.

The *C*_*POxC*_ was measured by adopting the potassium permanganate oxidation method [29]. The soil samples were dried under natural conditions and sieved to 0.25 mm, where the gravel and plant residue were discarded. A 0.5 g sieved soil sample was weighed and put into centrifuge tube with a volume of 50 mL, while adding 10 mL of 333 mmol L^-1^ potassium permanganate. The potassium permanganate supernatant was obtained after centrifuge tubes were shaken at 300 revolutions per minute for 1 hour and centrifuged at 4000 revolutions per minute for 5 minutes. In addition, 200 μL potassium permanganate supernatant was diluted with 100 mL of deionized water at a 1:500 dilution ratio. The concentration of potassium permanganate diluent was determined using a spectrophotometer at a wavelength of 565 nm. Finally, the mass fraction of *POxC* was calculated by the variation in the potassium permanganate concentration (g kg^-1^, which means the POxC content of 1 kg dried soil).

The *C*_*POC*_ was measured by using the sodium hexametaphosphate extraction method [30]. The soil samples were dried under natural conditions and sieved to 2 mm, where the gravel and plant residue were discarded. The sieved soil sample of 10 g was dispersed in 30 mL of 5 g L^-1^ sodium hexametaphosphate by shaking for 18 hours on a reciprocal shaker. The dispersed soil sample was passed through a 53 μm sieve by rinsing with distilled water until the filtrate was colorless and free of soil impurities. The part of the soil that did not pass through the 53 μm sieve was the particulate soil, and the other part was the mineral-associated soil. The particulate soil was collected and transferred to a preweighed aluminum box and then dried at 60°C until the weight was constant. The proportion of particulate and mineral-associated soil in the weighed soil sample (10 g) was calculated using Eqs (1) and (2), respectively. The dried particulate soil was sieved to 0.25 mm and was used to determine the SOC concentration in particulate soil. Then, the POC and MOC concentrations of the weighed soil sample were calculated using Eqs (3) and (4), respectively.

$$w_{poc}=\frac{w_{1}}{w_{0}}\times 100\%$$ (1)

$$w_{moc}=1-w_{poc}$$ (2)

where, *w*_*poc*_ and *w*_*moc*_ are the proportions of particulate soil and mineral-associated soil in the weighed soil sample, respectively, (%). *w*_*0*_ is the weight of the soil sample to be determined (10 g), and *w*_*1*_ is the weight of dried particulate soil (g).

$$c_{t-poc}=c_{poc}\times w_{poc}$$ (3)

$$c_{t-moc}=c_{t-soc}-c_{t-poc}$$ (4)

where, *C*_*t-poc*_ is the POC concentration (g kg^-1^), *C*_*t-moc*_ is the MOC concentration of the weighed soil sample (g kg^-1^), *C*_*poc*_ is the organic carbon concentration of the particulate soil sample (g kg^-1^), and *C*_*t-soc*_ is the total SOC concentration of the soil sample (g kg^-1^).

The SOC stock at each soil depth “*i*” (“*i”* is assigned for the values which represents 0–10, 10–20, 20–30, 30–40 and 40–50 cm, respectively) was calculated using Eqs (5) and (6):

$$M_{soil,i}=\rho_{b,i}\times T_{i}\times 10000$$ (5)

$${Stock}_{i}=M_{soil,i}\times {conc}_{i}\times 0.001$$ (6)

where, *M*_*soil*,*i*_ is the soil mass at depth “*i*” (Mg ha^-1^); *ρ*_*b*,*i*_ is the soil bulk density at depth “*i*” (g cm^-3^), the unit is converted to Mg m^-3^; *T*_*i*_ is the soil thickness at depth “*i*” (m); 10000 is the conversion coefficient of the area unit from m^2^ to ha^-1^ (m^2^ ha^-1^); *Stock*_*i*_ is the SOC stock at depth “*i*” (Mg ha^-1^); *conc*_*i*_ is the SOC concentration at depth “*i*” (g kg^-1^); 0.001 is the conversion coefficient of mass unit from kg to Mg (Mg kg^-1^).

Additionally, the SOC stock in different soil profile (i.e., 0–10, 0–20, 0–30, 0–40 and 0–50 cm) was estimated using the mass-equivalent method [31], and calculated using Eq (7):

$$Stock=[\sum_{i=1}^{n}M_{soil,i}\times {conc}_{i}+(\sum_{i=1}^{n}M_{o,i}-\sum_{i=1}^{n}M_{soil,i})\times {conc}_{i+1}]\times 0.001$$ (7)

where, the stock is the mass-equivalent stock of SOC (Mg ha^-1^) to a certain depth; *M*_*soil*,*i*_ and *i* represent the same value those in Eqs (5) and (6); $\sum_{i=1}^{n}M_{soil,i}$ is the summed soil mass (Mg ha^-1^) from depth “1” to depth “*n*”; $\sum_{i=1}^{n}M_{o,i}$ is the greatest summed soil mass (Mg ha^-1^) from depth “1” to depth “*n*” among three treatments; *conc_i_* and *conc*_*i*+1_ are the SOC concentration (g kg^-1^) at depth “*i*” and depth “*i+1*”, respectively; and 0.001 is the conversion coefficient of mass unit from kg to Mg (Mg kg^-1^).

### Statistical analysis

One-way analysis of variance was used to assess the effects of tillage during the summer fallow season for all datasets using the SPSS 16.0 (SPSS Inc., USA) software package. The mean differences among different treatments were separated by the new multiple range test (Duncan) at *P* < 0.05 for the measured soil variables. The relationships between soil bulk density, gravimetric water content, volumetric moisture content, total porosity, air-filled porosity, and capillary porosity were analyzed using Pearson correlation analysis.

## Results

### Soil organic carbon fractions

**Fig 1:**
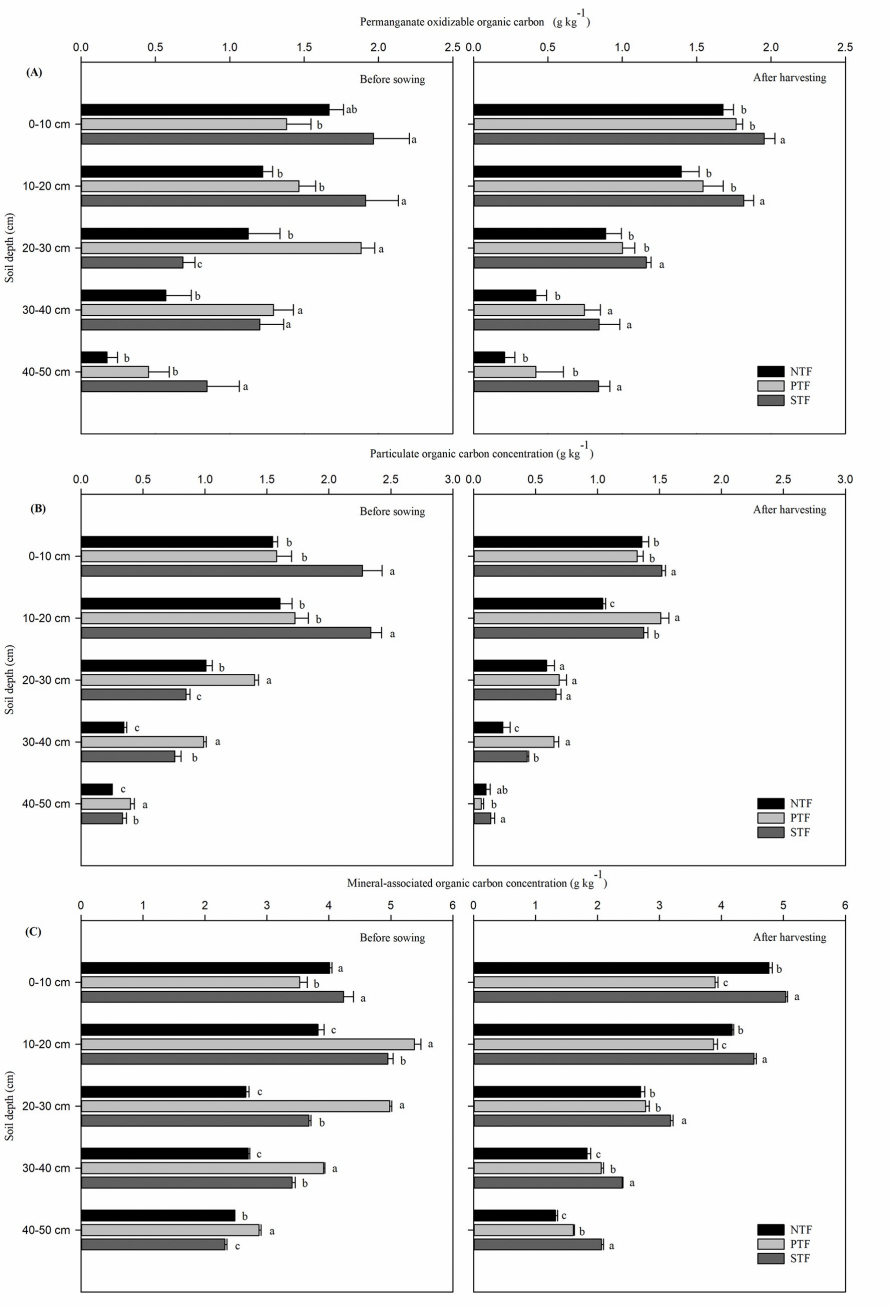
Soil organic carbon fractions: (A) permanganate-oxidizable organic carbon; (B) particulate organic carbon; (C) mineral-associated organic carbon under different tillage practices in the 0–50 cm profile before sowing and after harvesting winter-wheat. NTF: No tillage during the summer fallow period, PTF: Plow tillage during the summer fallow period, and STF: Subsoiling during the summer fallow period. The lowercase letters indicate statistical difference among different treatments at *P* < 0.05.

At the 0–10 cm soil depth, the POxC concentration under STF was highest both before sowing and after harvesting (Fig 1A). Specifically, the POxC concentration under STF increased significantly by 42.3% compared with that under PTF before sowing and increased significantly by 16.4% and 10.7% compared with those under NTF and PTF after harvesting, respectively (*P* < 0.05). At the 10–20 and 40–50 cm depths, higher POxC concentrations were also observed under STF than under NTF and PTF both before sowing and after harvesting (*P* < 0.05). At the 20–30 cm soil depth, the POxC concentration under STF was significantly lower than that under PTF and NTF before sowing, while after harvesting, the POxC concentration under STF was significantly higher than that under PTF and NTF by 30.8% and 15.9%, respectively (*P* < 0.05). At the 30–40 cm soil depth, the POxC concentrations before sowing and after harvesting were significantly higher under STF and PTF than under NTF (*P* < 0.05); however, those under STF were not significantly different from those under PTF.

At the 0–10 cm soil depth, compared to that under PTF and NTF, the POC concentration under STF was significantly higher by 43.9–46.9% and 11.7–14.9% before sowing and after harvesting, respectively (*P* < 0.05), and no significant difference was observed between PTF and NTF (Fig 1B). At the 10–20 cm depth, the POC concentration before sowing showed a similar trend as that at the 0–10 cm depth. The POC concentrations at the10-20 cm depth after harvesting and at the 20–30 cm and 30–40 cm depths before sowing and after harvesting were higher under PTF than that under the other treatments. At the 40–50 cm soil depth, the POC concentration before sowing showed the same trend as that at the 30–40 cm soil depth. After harvesting, the POC concentration under STF was significantly higher than that under PTF (*P* < 0.05); however, the POC concentration under NTF was not significantly different from that under STF.

Before sowing, the MOC concentration at the 0–10 cm depth was 12.0% and 16.7% less under PTF than under NTF and STF, respectively (Fig 1C). At the 10–20, 20–30, 30–40 and 40–50 cm soil depths, the MOC concentrations under PTF were significantly higher than those under STF and NTF (*P* < 0.05). After harvesting, compared to that under PTF and NTF, the MOC concentration under STF was significantly higher by 5.6–29.2%, 8.4–16.9%, 14.5–17.9%, 16.4–31.0% and 28.4–56.4% at the 0–10, 10–20, 20–30, 30–40 and 40–50 cm soil depths, respectively (*P* < 0.05).

### Soil organic carbon concentration

**Fig 2:**
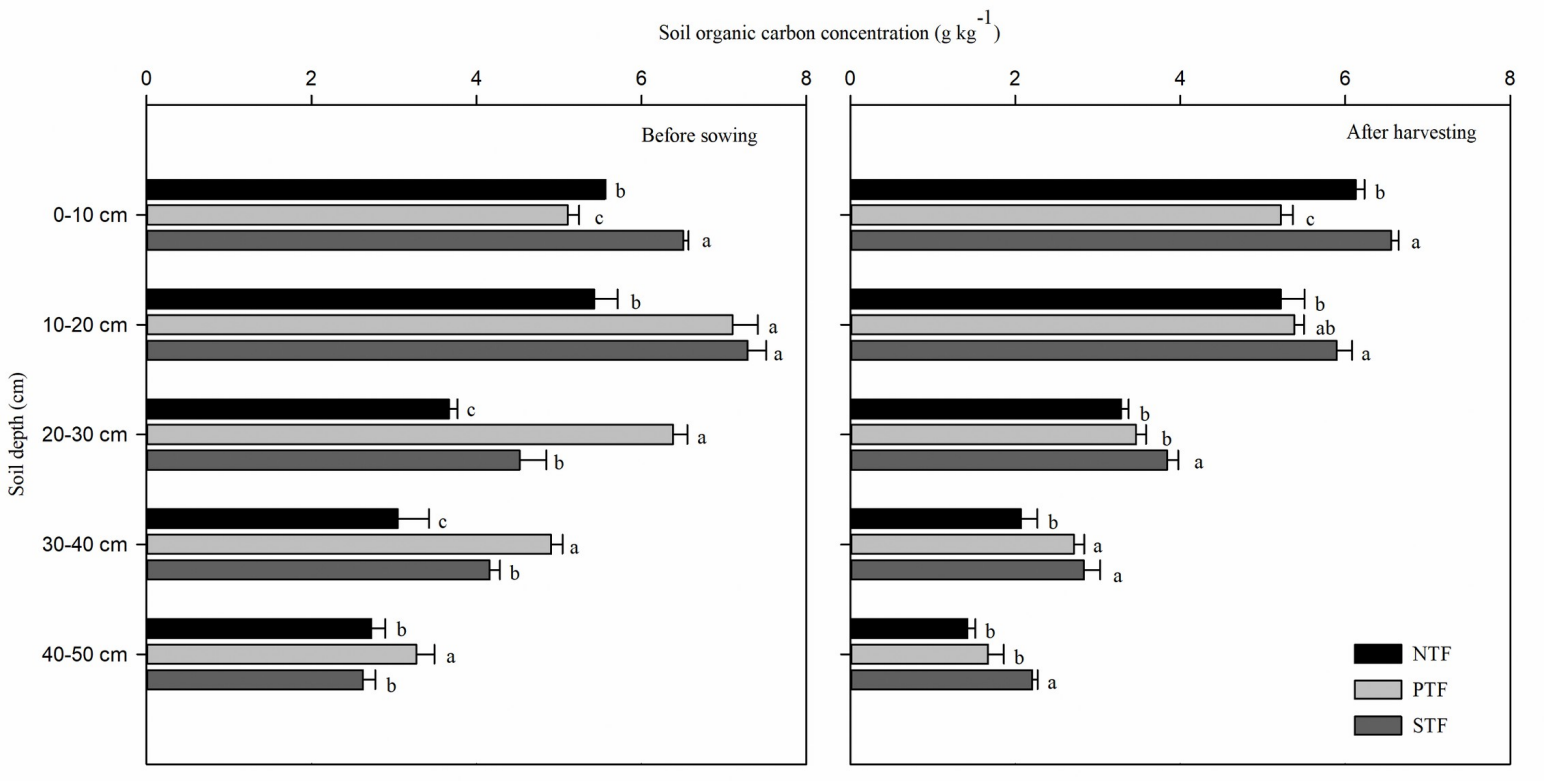
Soil organic carbon concentration under different tillage practices in the 0–50 cm soil profile before sowing and after harvesting winter-wheat. NTF: No tillage during the summer fallow period, PTF: Plow tillage during the summer fallow period, and STF: Subsoiling during the summer fallow period. The lowercase letters indicate statistical difference among different treatments at *P* < 0.05.

At the 0–10 and 10–20 cm soil depths, the SOC concentrations under STF both before sowing and after harvesting were significantly higher than those under NTF (*P* < 0.05) (Fig 2). At depths of 20–30 and 30–40 cm, the SOC concentrations before sowing ranked as PTF > STF > NTF with significant difference (*P* < 0.05). However, the highest SOC concentrations at the 20–30, 30–40 and 40–50 cm soil depths were observed under STF after harvesting.

### Soil organic carbon stock

**Fig 3:**
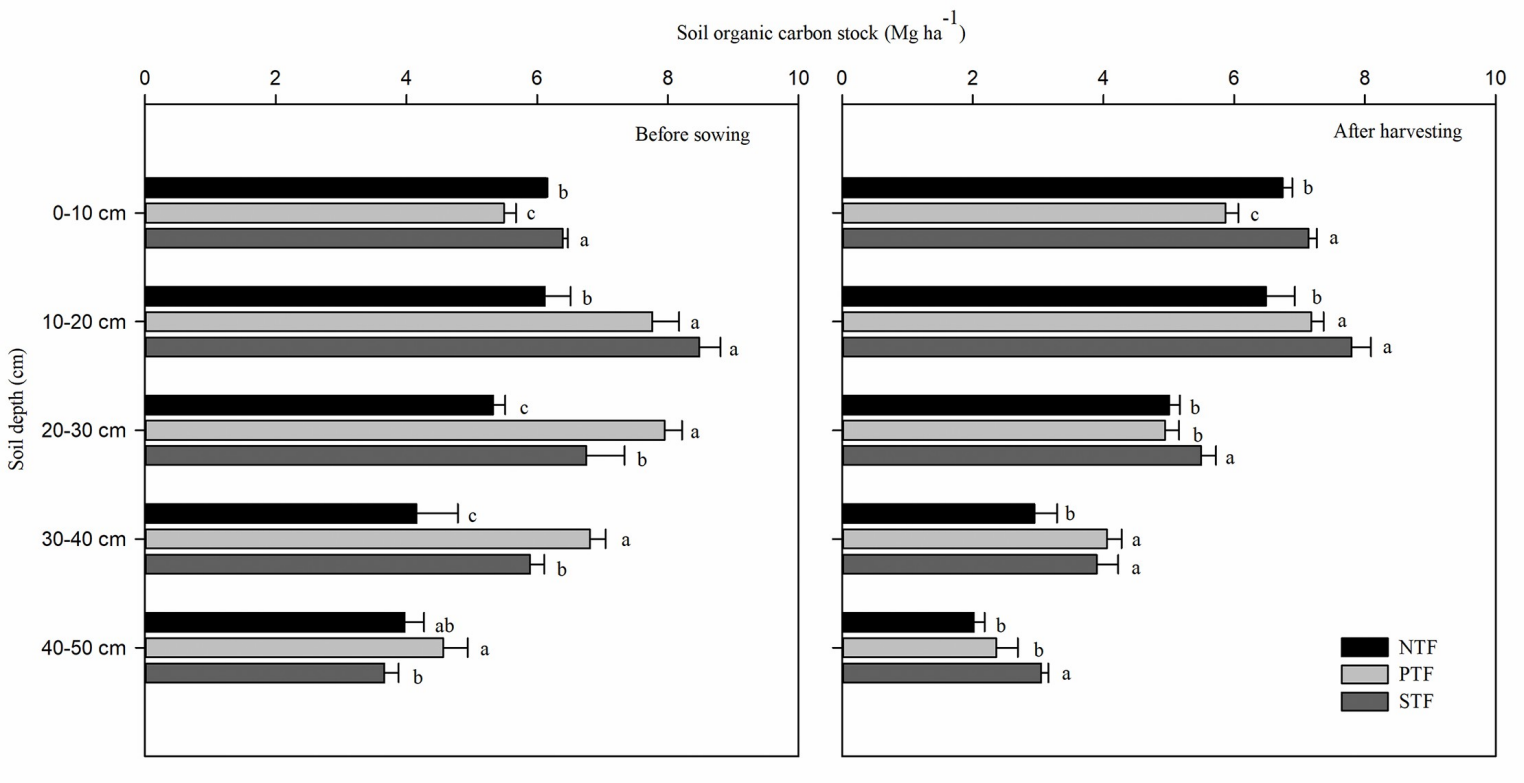
Soil organic carbon stocks under different tillage practices in the 0–50 cm profile before sowing and after harvesting. NTF: No tillage during the summer fallow period, PTF: Plow tillage during the summer fallow period, and STF: Subsoiling during the summer fallow period. The lowercase letters indicate statistical difference among different treatments at *P* < 0.05.

At the 0–10 cm soil depth, the SOC stocks under STF before sowing and after harvesting were significantly higher than those under PTF and NTF, and the SOC stocks under NTF were also significantly higher than those under PTF (*P* < 0.05) (Fig 3). At the 10–20 cm soil depth, significantly higher SOC stocks were observed under STF and PTF than under NTF before sowing and after harvesting (*P* < 0.05), and STF was not significantly different from PTF. At the 20–30, 30–40 and 40–50 cm soil depths, the SOC stocks under PTF before sowing were significantly higher than those under STF, and those under STF were significantly higher than those under NTF at the 20–30 and 30–40 cm soil depths (*P* < 0.05). After harvesting, the SOC stocks under STF were significantly higher than those under NTF at the 20–30, 30–40 and 40–50 cm soil depths, and significantly higher than those under PTF at 20–30 and 40–50 cm soil depths (*P* < 0.05).

Before sowing, the SOC stocks in the 0–10 cm and 0–20 cm soil profiles were significantly larger under STF than those under NTF and PTF (Table 1). Within the 0–30 cm, 0–40 cm and 0–50 cm soil profiles, the SOC stocks under STF and PTF were significantly larger than those under NTF (*P* < 0.05), whereas, no significant differences were observed between STF and PTF. After harvesting, the SOC stocks in the 0–10 cm, 0–20 cm, 0–30 cm, 0–40 cm and 0–50 cm soil profiles were significantly larger under STF than under PTF and NTF (*P* < 0.05).

**Table 1:** Soil organic carbon stock in different soil profile under different tillage practices for dryland winter-wheat (Mg ha^-1^).

Sampling time	Treatment	Soil profile				
		0–10 cm	0–20 cm	0–30 cm	0–40 cm	0–50 cm
**Before sowing**	**NT**	6.14±0.02b	12.26±0.37c	17.58±0.39b	21.74±0.24b	25.71±0.39b
	**PT**	5.49±0.18c	13.26±0.47b	21.21±0.67a	28.01±0.82a	32.57±0.99a
	**ST**	6.39±0.08a	14.87±0.26a	21.62±0.85a	27.51±1.06a	31.17±0.91a
**After harvest**	**NT**	6.86±0.15b	13.60±0.51b	18.27±0.61b	21.30±0.96b	23.31±1.10b
	**PT**	5.87±0.20c	13.04±0.37b	17.99±0.49b	22.04±0.69b	24.40±0.98b
	**ST**	7.35±0.13a	15.11±0.43a	20.55±0.64a	24.68±0.54a	27.79±0.44a

Note: NTF: No tillage during the summer fallow period, PTF: Plow tillage during the summer fallow period, and STF: Subsoiling during the summer fallow period. The lowercase letters indicate statistical difference among different treatments at P<0.05.

Pearson correlation was determined between the soil physical and chemical properties, enzyme activity and indicators of the SOC pool. The concentrations of SOC and its fractions were significantly negatively correlated with bulk density (*P* < 0.01) but positively correlated with gravimetric moisture, total porosity, β-glucosidase, and urease activities (*P* < 0.05) (Table 2).

**Table 2:** The relationship of SOC and its fractions to soil physical and chemical properties.

Soil properties	SOC	POxC	POC	MOC
Bulk density	-0.793**	-0.770**	-0.823**	-0.744**
Gravimetric moisture content	0.509**	0.557**	0.413*	0.530**
Volumetric moisture content	0.164	0.225	0.06	0.207
Total porosity	0.769**	0.746**	0.800**	0.718**
Air-filled porosity	0.508**	0.458*	0.587**	0.447*
Capillary porosity	0.248	0.277	0.174	0.261
Heavy fraction soil	-0.806**	-0.733**	-0.829**	-0.756**
Light fraction soil	0.806**	0.733**	0.829**	0.756**
Available N	0.401*	0.512**	0.491**	0.347
Available P	0.795**	0.826**	0.821**	0.761**
Available K	0.691**	0.738**	0.636**	0.680**
β-glucosidase	0.892**	0.896**	0.869**	0.859**
Urease	0.639**	0.474**	0.665**	0.611**
MWD of MSA	-0.388*	-0.233	-0.381*	-0.376*
GMD of MSA	-0.395*	-0.247	-0.393*	-0.381*
Fractal dimension of MSA	0.268	0.224	0.252	0.269
MWD of WSA	-0.459*	-0.503**	-0.453*	-0.451*
GMD of WSA	-0.416*	-0.262	-0.430*	-0.409*
Fractal dimension of WSA	0.175	0.220	0.202	0.162
Water stable aggregate rate	-0.183	-0.236	-0.182	-0.178
PAD_0.25_	0.175	0.230	0.172	0.172
Stable aggregate index	0.212	0.234	0.223	0.201

Note: SOC is total soil organic carbon, POxC is permanganate-oxidizable organic carbon, POC is particulate organic carbon, MOC is mineral-associated organic carbon, MWD: Mean weight diameter, GMD: Geometric Mean Diameter, MSA: Mechanical stable aggregate, WSA is water stable aggregate, PAD_0.25_ is percentage of aggregate destruction.

*and ** indicate that person correlation is significant at *P* < 0.05 and *P* < 0.01 level, respectively.

## Discussion

In current study, the experimental station is located in the rainfed farmfields on the Loess Plateau in China. Precipitation is the sole source of water for the growth of winter-wheat in this area. And approximately 70–80% of the annual precipitation occurs during the summer fallow period of the rainfed winter-wheat field, which does not coincide with the water demand period for winter-wheat growth. In order to solve the problem, the practice, which refers to soil deep tillage, residue management and organic fertilizer application in advance during the summer fallow season, has been proposed. This practice can significantly conserve precipitation in the soil during the summer fallow season and enhance the yields of winter-wheat [28]. However, the effect of this practice SOC sequestration has not been figured out clearly. It is significant to clarify the effect of SOC sequestration under this practice for further scientific management of the SOC pool and improvement of the crop productivity.

### Effect of tillage practices on the concentration of SOC fractions

As a labile SOC fraction, POxC is sensitive to farm management measures. In this study, in comparison to PTF, STF significantly increased the POxC concentrations at the 0–10 and 10–20 cm soil depths before sowing (*P* < 0.05). The result accorded with the finding reported by Hu et al. [32] that reducing tillage frequency and intensity could increase labile SOC concentration by improving soil microbial community compared with conventional plow tillage. Another reasonable explanation for this result is that the crop residue provided crucial carbon supplementation of labile SOC fractions before sowing. A large amount of crop residue under PTF was turned into 25–30 cm soil under PTF, while under STF, it was incorporated into the 0–35 cm soil depth. The crop residue under STF distributed in the 0–20 cm soil layer was larger than that under PTF, which provided more carbon supplementation for microbes, so that the POxC concentration under STF increased. Similarly, the POxC concentration at the 20–30 cm soil depth under PTF was significantly higher than that under STF due to the larger amount of crop residue at this depth (*P* < 0.05). In addition, the POxC concentration under STF at the 10–20 cm soil depth was significantly higher than that under NTF (*P* < 0.05). The possible reason for this result may be that the subsoiling adopted in the summer fallow in the STF plots decreased soil bulk density, increased soil porosity, and improved soil permeability by loosening soil, which could accelerate decomposition of crop residues, even though rotary tillage at the 10–15 cm soil depth was implemented for all tillage plots, and the crop residue under NTF was also incorporated at the 0–20 cm soil depth.

However, the POxC concentration at the 0–10, 10–20 and 20–30 cm soil depths under PTF after harvesting was significantly lower than that under STF (*P* < 0.05). This result may have been due to the intensive disturbances to the soil under PTF. The soil structure was destroyed by turning over soil under PTF, which accelerated the mineralization of POxC. Furthermore, in comparison with NTF, STF significantly increased POxC concentration at the 30–40 and 40–50 cm soil depths before sowing and after harvesting (*P* < 0.05). The main explanation for this finding may be due to the difference in the distribution of crop roots. Likewise, Wright et al. [33] explained that SOC sequestrated at deeper depth may be more dependent on crop root carbon deposition than aboveground residues, as residue inputs can be 3.5 times greater for roots than stover. Previous studies have illustrated that subsoiling could promote crop root growth to deeper tilled depths and even deeper soil by breaking up dense soil layers, improving soil physical structure (e.g., increased soil total porosity) [34]. In this study, a 30–35 cm tillage depth under STF may promote deeper growth of wheat roots, yet the root system under NTF concentrated on the tillage zone due to higher soil bulk density and resistance [35]. Therefore, the difference of carbon input from crop roots directly affected the change of POxC concentration.

The POC, combined with sand grains of 53–2000 μm, is also a variable SOC fraction. In comparison to PTF, STF significantly increased the POC concentration at the 0–10 and 10–20 cm, whereas the POC concentration significantly decreased at the 30–40 and 40–50 cm soil depths before sowing (*P* < 0.05). The decrease in POC concentration occurred at upper depth under PTF was consistent with the result reported by Dou et al. [36] due to more rapid decomposition. This result likely attributed to more soil disturbance and large soil aggregates destruction under PTF, which caused POC protected by aggregates to be exposed to air and accelerated its decomposition. Similarly, due to soil disturbance with a 30–35 cm tillage depth with STF, the POC concentration at corresponding and deeper soil depths (30–40 and 40–50 cm) was significantly lower than that under PTF (*P* < 0.05). In addition, In comparison with STF and NTF, PTF significantly increased the POC concentration at the 20–30 cm soil depth. The result may be associated with sufficient carbon input sourced from crop residues. The adoption of PTF resulted in large amount of crop residues being turned into the 25–30 soil depth, which was beneficial to the formation of aggregates.

The MOC, combined with clay and silt grains smaller than 53 μm, is a recalcitrant SOC fraction used to evaluate SOC sequestration. In this study, the MOC concentration under PTF below a 10 cm soil depth was significantly higher than those under STF and NTF (*P* < 0.05). This result may be attributed to the fact that the original aggregate particles were destroyed by turning over soil under PTF, and the clay-silt complex content in the soil that was less than 53 μm increased. The mineral particles were combined with SOC to promote the formation of MOC [37]. However, the MOC concentration under PTF showed increasing trend only in the short term because the strong disturbance to soil may have accelerated MOC mineralization, so that the MOC concentration under PTF was significantly lower than that under STF after harvesting. Adoption of STF improved soil permeability rather than damaging soil structure, and promoted increased greater growth of the crop root system, which provided sufficient crop residue and increased MOC concentration after harvesting [35].

### Effect of tillage practices on SOC concentrations and stocks

The SOC concentration at the 0–10 cm soil depth before sowing was significantly larger under STF and NTF than under PTF (*P* < 0.05) and was similar to the results reported by previous researchers [38,39]. The lower SOC concentration in our study under PTF might have been due to a large volume of crop residues being turned into the deeper layer (25–30 cm) through the summer tillage practice. Although tillage depth under STF increased to 30–40 cm, fewer crop residues were incorporated at this depth because the soil was loosened rather than turned over. Therefore, the higher SOC concentration under NTF and STF than that under PTF at 0–10 cm may have been due to sufficient carbon inputs from crop residues [40].

However, in comparison with STF, NTF significantly decreased the SOC concentration at the 10–20, 20–30 and 30–40 cm soil depths before sowing (*P* < 0.05). Similarly, Qian et al. [41] suggested that the conversion from abandoned to cultivated lands reduced the stability of soil aggregate, accelerated the fragmentation of soil aggregate, decreased the proportion of macro-aggregates, and increased the proportion of micro-aggregates. All these changes stabilized the maintenance of SOC of micro-aggregate and accelerated the accumulation of the SOC pool. The finding also provided evidence for the result that there was a significant negative correlation between SOC concentrations and mean weight diameter, geometric mean diameter of aggregate in our study. Another possible reason for this result is that there was little carbon input from crop residue under the NTF treatment, and the continuous SOC decomposition resulted in the decrease in the SOC concentration [42]. Modak et al. [43] also emphasized the importance of crop residue retention under reduced tillage to deep SOC sequestration. In summary, carbon source input and carbon outputs (i.e., mineralization) are important processes that affect SOC sequestration. However, there is little research on the contribution of crop residues and root systems to SOC and its fraction formation in different crop-growing seasons. Therefore, additional research will be conducted adopting the isotope labeling method [44] in future work. Furthermore, it will be important to study the mineralization characteristics of SOC under the three tillage practices during summer fallow period in the future.

In addition, the SOC concentrations at the 0–10, 10–20, 20–30, 30–40 and 40–50 cm soil depths after harvesting were significantly higher under STF than under NTF (*P* < 0.05). A plausible explanation for this result is the improvement in soil porosity and permeability as a consequence of the looser soil and lower soil bulk density under STF than under NTF. These factors can facilitate the activities of microbial activity and carbon cycle enzymes, resulting in an increase in the SOC concentration [45,46]. In addition, the evidence in our study showed that the SOC concentration was positively related to β-glucosidase and urease activities, but negatively related to bulk density. Moreover, improved soil porosity and air permeability under STF were conducive to the development of crop root systems, which provide sufficient carbon sources for the accumulation of SOC concentrations [34].

In our study, the SOC stock was calculated by adopting equivalent soil mass measurements. The maximum soil mass among the three tillage treatments at each soil depth was selected as a reference soil mass, therefore, the SOC stock of each treatment was only related to the SOC concentration. The SOC stocks under each treatment (NTF, STF, and PTF) after harvesting were lower than those before sowing in the 0–50 cm soil profile in this study, which was different from previous results where long-term continuous no tillage was beneficial to increasing SOC stocks [9,10]. By converting plow tillage to no tillage, the SOC stock may reach a new balance after 15–30 years in different regions [47]. Consequently, tillage year had a great impact on SOC sequestration, and the time to a new balance varied from region to region. This variation may have been related to the relatively shorter duration of the tillage experiment from 2011–2015. It is necessary to increase the tillage years to observe the effects of different tillage practices on SOC sequestration. Moreover, many environmental factors, such as climatic conditions, topography, vegetation types, biodiversity, soil texture and straw input could affect SOC stocks [48]. Thus, further expanding the study in different ecological regions, farming systems, vegetation types and soil conditions is of great significance for evaluating the impact of tillage practices on SOC stocks.

## Conclusions

Tillage practices significantly affect SOC and its fractions (POxC, POC and MOC). The SOC concentrations before sowing and after harvesting were significantly higher by 7.0–36.9% under STF than under NTF at the 0–10, 10–20, 20–30 and 30–40 cm soil depths. In comparison with PTF and NTF, STF significantly increased POxC and MOC concentrations at the 0–10, 10–20, 20–30, 30–40 and 40–50 cm soil depths after harvesting (*P* < 0.05). Among the three tillage treatments, STF had the highest SOC concentrations at the 0–10, 10–20, 20–30, 30–40 and 40–50 cm soil depths after harvesting. The SOC stocks at the 0–10, 10–20, 20–30 and 30–40 cm soil depths under STF were significantly higher by 4.1–41.9% and 5.9–32.4% than those under NTF before sowing and after harvesting, respectively (*P* < 0.05). Furthermore, in comparison with PTF and NTF, STF significantly increased the SOC stocks in the 0–10, 0–20, 0–30, 0–40 and 0–50 cm soil profiles after harvesting (*P* < 0.05). Overall, STF could be a useful practice to improve soil quality and enhance SOC sequestration in rainfed winter-wheat fields on the eastern Loess Plateau of China.

## Supporting information

S1 Fig Raw data of POxC concentration analysis. (XLSX)

S2 Fig Raw data of POC and MOC concentration analysis. (XLSX)

S3 Fig Raw data of SOC concentration analysis. (XLSX)

S1 Table Raw data of SOC stock analysis. (XLSX)

S2 Table Analysis process of the relationship of SOC and its fractions to soil physical and chemical properties. (XML)
